# How efficient coding of binocular disparity statistics in the primary visual cortex influences eye rotation strategy

**DOI:** 10.1186/1471-2202-14-S1-O7

**Published:** 2013-07-08

**Authors:** Sarah E Marzen, Joel Zylberberg, Michael R DeWeese

**Affiliations:** 1 Department of Physics, U. C. Berkeley, Berkeley, CA 94708, USA; 2 Department of Applied Mathematics, University of Washington, Seattle, WA 98195, USA; 3 Helen Wills Neuroscience Institute, U.C. Berkeley, Berkeley, CA 94708, USA

## 

Stereopsis, the ability to perceive depth, is crucial for detecting camouflaged objects and for performing tasks that require estimating distance. Understanding how our brain can so quickly estimate depth from the slightly different images from our left and right eyes, the difference of which is called binocular disparity, is still a major unsolved problem in vision research. Several previous computational studies of binocular disparity have found evidence suggesting that various properties of the primary visual cortex (V1) allow V1 to optimally process natural binocular disparity statistics. In particular, the efficient coding hypothesis suggests that the disparity tuning of V1 binocular neurons should reflect the natural range of disparities [1,2]; the cortical wiring hypothesis suggests that the orientation of ocular dominance stripes should follow the binocular disparity map [3]; and visuomotor optimization theory and the efficient coding hypothesis suggests that eye rotation strategy should be chosen to minimize binocular disparity and motor inefficiency [4,5].

In order to understand how natural binocular disparity statistics might be efficiently coded by the primary visual cortex, we constructed a simulation that links natural scene statistics to eye movements to connections between monocular neurons in V1. We generate a three-dimensional visual environment with two-point statistics and observer-object distance distribution similar to that of a natural environment; a virtual observer rotates her eyes according to the binocular version of Listing's Law to fixate on either edges of objects, centers of objects, or randomly chosen points on objects; and the resulting binocular disparities are mapped to V1 by a Schwartz conformal map fit to physiological data. The effects of two-point statistics, primary Listing's plane exorotation angle, Listing's Law coefficient, and fixation strategy can be revealed using this simulation in ways that would be very difficult (if not impossible) with experiments. For instance, to measure the effect of two-point statistics on binocular disparity statistics using this simulation, we compare the binocular disparity statistics in our more complicated three-dimensional visual environment to a visual environment with the same observer-object distance distribution but no pixel-pixel correlations.

Our simulations show that the predicted ocular dominance stripe orientations and stereoscopic search zones are largely insensitive to two-point statistics of the visual environment, but very sensitive to interocular distance and eye rotation strategy. Finally, our more careful treatment of oculomotor strategy and visual environment shows that physiological oculomotor strategy cannot be explained by current visuomotor optimization theory[5]. A modified visuomotor optimization theory will likely require a better understanding of stereopsis-related processing in the visual cortex.
